# Development of a Soil Moisture Prediction Model Based on Recurrent Neural Network Long Short-Term Memory (RNN-LSTM) in Soybean Cultivation

**DOI:** 10.3390/s23041976

**Published:** 2023-02-10

**Authors:** Soo-Hwan Park, Bo-Young Lee, Min-Jee Kim, Wangyu Sang, Myung Chul Seo, Jae-Kyeong Baek, Jae E Yang, Changyeun Mo

**Affiliations:** 1 Interdisciplinary Program in Smart Agriculure, Kangwon National University, Chuncheon 24341, Republic of Korea; 2 Agriculture and Life Sciences Research Institute, Kangwon National University, Chuncheon 24341, Republic of Korea; 3 Divison of Crop Physiology and Production, National Institute of Crop Science, Rural Development Administration, Hyoksin-ro 181, Iseo-myeon, Wanju-gun 55365, Republic of Korea; 4 Department of Natural Resources and Environmental Sciences, Kangwon National University, Chuncheon 24341, Republic of Korea

**Keywords:** smart farming, time series analysis, soil moisture, deep learning, RNN-LSTM

## Abstract

Due to climate change, soil moisture may increase, and outflows could become more frequent, which will have a considerable impact on crop growth. Crops are affected by soil moisture; thus, soil moisture prediction is necessary for irrigating at an appropriate time according to weather changes. Therefore, the aim of this study is to develop a future soil moisture (SM) prediction model to determine whether to conduct irrigation according to changes in soil moisture due to weather conditions. Sensors were used to measure soil moisture and soil temperature at a depth of 10 cm, 20 cm, and 30 cm from the topsoil. The combination of optimal variables was investigated using soil moisture and soil temperature at depths between 10 cm and 30 cm and weather data as input variables. The recurrent neural network long short-term memory (RNN-LSTM) models for predicting SM was developed using time series data. The loss and the coefficient of determination (R^2^) values were used as indicators for evaluating the model performance and two verification datasets were used to test various conditions. The best model performance for 10 cm depth was an R^2^ of 0.999, a loss of 0.022, and a validation loss of 0.105, and the best results for 20 cm and 30 cm depths were an R^2^ of 0.999, a loss of 0.016, and a validation loss of 0.098 and an R^2^ of 0.956, a loss of 0.057, and a validation loss of 2.883, respectively. The RNN-LSTM model was used to confirm the SM predictability in soybean arable land and could be applied to supply the appropriate moisture needed for crop growth. The results of this study show that a soil moisture prediction model based on time-series weather data can help determine the appropriate amount of irrigation required for crop cultivation.

## 1. Introduction

Currently, soybeans are one of the most economically important bio-based crops; they are also used as a major source of protein and oil for food and animal feed [1]. Soybeans are summer crops with high water requirements; therefore, considerable water is needed to increase soybean yield [2]. The water used by plants is mainly soil moisture, indicating the amount of water that crops can use during the growing season and is very important for agriculture [3]. The growth and degradation of soil moisture directly affects water consumption and crop growth and are important indicators of drought resistance, flood control, and precision irrigation determination in agriculture production [4,5,6]. In addition, a lack of moisture in the soil causes stress in crops, which hinders the growth of plants [7]. In South Korea, soil moisture fluctuates due to weather changes, such as rainy seasons and droughts from June to October (i.e., the period of time when soybeans are grown); hence, soil moisture plays a significant role in crop production under cultivation conditions that depend on rainfall. In addition, the investigated relationship between crop yield and soil moisture shows that soil moisture control plays an important role in increasing crop yield [8,9,10]. Accurate prediction of soil moisture is crucial for properly managing agricultural water resources and increasing crop yields.

Natural disasters, such as floods and droughts, are causing a decrease in crop yields globally, while crop growth is directly affected by various environmental factors, such as soil moisture, temperature, and humidity. As precipitation is directly involved in soil moisture in open-field crops, soil moisture prediction is required for efficient cultivation due to large variations in soil moisture (SM) [11,12,13].

To predict future SM, current SM must be accurately measured. Several studies have been conducted in Korea and abroad to accurately measure current soil moisture. A representative method for directly measuring soil moisture uses gravimetric analysis and dielectric constant sensors. In the case of the gravimetric method, the weight of moisture present in the soil is measured by collecting soil samples; however, the sample is exposed to the external environment and continuous measurement is difficult. The dielectric constant type of SM measurement mainly uses a frequency domain reflectometry (FDR) and time domain reflectometry (TDR) sensor. As the dielectric constant sensor continuously measures the soil moisture at each point and provides fast and reliable measurement results, it is suitable for real-time monitoring of SM. Thus, a dielectric constant sensor was used to measure SM in the study presented in [14].

In agriculture, studies on soil moisture prediction using artificial intelligence have been conducted in various ways [15]. Recent studies have applied deep learning technology to overcome the problem of low prediction accuracy of soil moisture due to irregular and complex characteristics of soil [16,17]. Deep neural network regression (DNNR) has been used to predict average soil moisture per day [18]. The soil moisture was predicted by applying an artificial neural network (ANN), convolution neural network (CNN), deep belief network (DBN), autoencoders (AEs), extreme learning machines (ELMs), and long short-term memory (LSTM) techniques [19].

Among them, the recurrent neural network (RNN) has been known to be effective in data processing with sequential data such as time series data [20]. However, early RNNs consisted of simple algorithms, which constantly encountered problems such as gradient vanishing during training; this resulted in RNNs lacking practicality for long sequences [21]. Therefore, the method of long short-term memory (LSTM) is proposed to solve the vanishing gradient problem for long sequences [22]. To address slope congestion and extinction problems, the LSTM adds a step in determining whether or not to pass the result value processed by the hidden layer when it moves to the next time point; i.e., inputs and outputs are opened or closed through each gate, and long-term dependencies are supplemented by solving slope congestion and extinction problems. Recently, a research study using the LSTM method for predicting soil temperature and moisture has been conducted, and its findings indicate the possibility of improving soil moisture prediction performance [23]. However, to improve the performance of the moisture content prediction model, selecting predictors is required; additionally, soil moisture is influenced by various factors such as precipitation, previous soil moisture, temperature, and humidity [24].

Therefore, the purpose of this study is to develop a circulation deep learning model to predict future SM using time series patterns of SM and weather data up to the forecasting point in soybean cultivation. In particular, the optimal factor for predicting future SM at three different soil depths (10 cm, 20 cm, and 30 cm) was identified using released weather data (temperature, relative humidity, and precipitation), and current soil temperature and moisture data of the cultivation environment. Lastly, an SM prediction model for each soil depth based on environmental factors was developed.

## 2. Materials and Methods

### 2.1. Data Acquisition and Measurement

**Figure 1:**
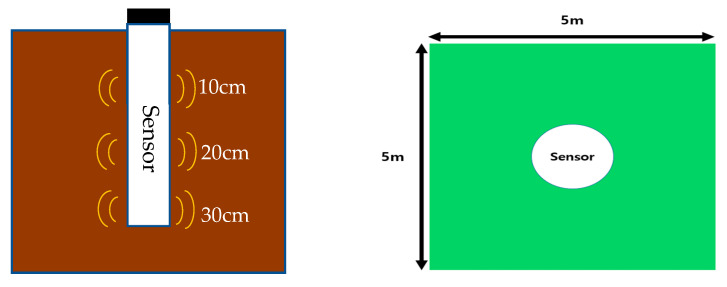
Soil sensor (Drill & Drop) (**Left** part) and measurement field (**Right** part).

Soil data (SM, soil temperature) and weather data (temperature, humidity, and precipitation) from 5 July 2020 to 4 October 2020 (i.e., period of time before harvesting soybeans) were obtained. A sensor installed at the center of the cultivation field (5 m × 5 m) collected SM and soil temperature (ST) data within approximately 1 m. The sensor used a TDR type soil sensor (SDI-12, Sentek Drill & Drop Probes, Stepney, Australia). The details of the soil sensor are presented in Table 1. The roots of soybean plants are mainly distributed at depths of 0–30 cm from the topsoil, and the number of roots rapidly decreases at depths exceeding 30 cm [25]. Therefore, the installed soil sensor measured the SM and temperature measurements at intervals of 10 min for each depth of 10, 20, and 30 cm from the soybean cultivation field in Iseo-myeon, Wanju-gun, Jeollabuk-do. The properties of the soil in the field were investigated and, on average, it had contents of sand, silt, and clay of 36.84%, 35.56%, and 27.60%, respectively. The soil was classified as clay loam by USDA soil classification. Clay loam accounts for about 42% of the total field soil in Korea and, according to research, it is the soil that has the highest effect on the increase in soybean yield due to soil changes [26]. In order to predict the future SM using minimal sensors, this study utilized publicly available environmental data. Weather data of air temperature (T), relative humidity (RH), and precipitation (P) were taken from the data released by the Rural Development Administration’s Agricultural Weather Information Service. These weather data were automatically measured as data provided by the Korean government, and their accuracies were more than 97.0%. The obtained weather data included T, RH, and P, and their values were measured at 10 min intervals (Figure 1).

### 2.2. LSTM Model for Predicting Soil Moisture

**Figure 2:**
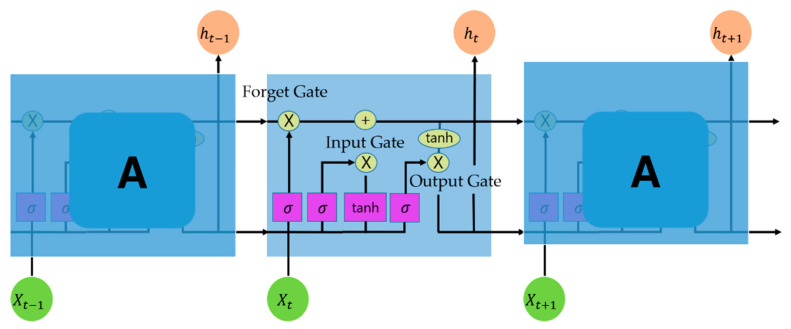
LSTM structure.

LSTM is a type of RNN model. The RNN structure is basically composed of three layers (input, hidden, and output), and the hidden layer is connected to previous data. However, the hidden layer of the RNN had a problem of long-term dependencies, in which only previous values were reflected in present data and the influence of past data decreased over time; thus, it was not practically applied in long sequences. To solve the long-term dependency problem, three gates (input, forget, and output) were created in the existing RNN and old information was passed through them [17]. The LSTM structure is shown in Figure 2. The cell outputs the output value *y_t_* at the current time point, the hidden state *h_t_* at the current time point using the input value *X_t_* at the current time point, and the hidden state *h_t_*_−1_ at the previous time point. The input and forget gates store necessary information from past and current information, and delete unnecessary information, while the output gate determines the output information using cell state information [27].

(1) $$f_{t}=\sigma (w_{fg}\cdot \left[h_{t-1},x_{t}\right]+b_{fg})$$

(2) $$i_{t}=\sigma (w_{ip}\cdot \left[h_{t-1},x_{t}\right]+b_{ip})$$

(3) $$c_{t}=tanh(w_{tcs}\cdot \left[h_{t-1},x_{t}\right]+b_{tsc})$$

(4) $$c_{t}=f_{t}sign\times c_{t-1}+{i_{t}sign\times c}_{t}$$

(5) $$o_{t}=\sigma (w_{op}\cdot \left[h_{t-1},x_{t}\right]+b_{op})$$

(6) $$h_{t}=o_{t}sign\times tanh_{t}(c_{t})$$

(7) $$y_{t}=W_{op}sign\times h_{t}+b_{op}$$

where *f_t_* is the forgotten information; *W_fg_* is the weight; *b_fg_* is the bias; *h_t_*_−1_ is the output value of the previous layer; *x_t_* is the input value of the current layer; σ determines the amount of discarded information in the forget gate; the range value of the σ function is (0, 1); *i_t_* is the input information; *w_ip_, b_ip_* are the weights and biases of the input gate; *𝑊_tcs_*, *𝑏_tsc_* are the weights and biases of the temporary cell state, which are the output values of the previous layer and the current layer; the range of the 𝑡𝑎nh function is (−1, 1); *c_t_* is the temporary cell state *w_op_*, and *b_op_* are the weights and biases of the output gate; and *o_t_* is controlled by the function.

The forget gate determines the precious $c_{t-1}$ storage state by inputting the previous hidden state value $h_{t-1}$ and the current input value $x_{t}$ into the sigmoid function (Equation (1)). The input gate is a combination of the previous and current layers; that is, the previous hidden state value and the new input value are inputted into sigmoid (Equation (2)) and (Equation (3)) to determine the expected new input value to be stored. The 𝐶_𝑡_ equation represents the value of the cell state calculated by the previous cell state, forgetting information, and temporary cell state. The $c_{t}$ multiplies the previous state value ($c_{t-1}$) by the forgetting gate to determine the $c_{t}$ of the current state by multiplying the holding degree value of the previous state value by the vector value for the input gate and the new input (Equation (4)). This is an implementation of combining historical and current data, which is a key feature of the LSTM model (Equations (3) and (4)). The output gate determines the output value by the sigmoidization of the previous hidden state value and the new input value (Equation (5)), and the current hidden value is determined by multiplying the value ($o_{t}$) of the output gate and the tanh value of the current $c_{t}$ (Equation (6)). In summary, the forgetting gate can reduce the long-term dependency problem with the logic of determining how much the previous memory cell value should be forgotten. The input and output gates determine the sizes of the new input value and output values, respectively. The final value $y_{t}$ is obtained by using the ouput output weight and the output bias (Equation (7)).

### 2.3. Conditions for Development of RNN-LSTM Model for Prediction of Soil Moisture

The SM prediction model is an algorithm that predicts SM after 10 min using 10 min interval data from 1 h before to the present using the RNN-LSTM based on TensorFlow (i.e., Python library). MinMaxScaler was used as a data preprocessing method for data entering the predictive model because it could overcome the scaling of software error data in the range between 0 and 1 in the simulation environment (Equation (8)). The standard activation functions are contractive almost everywhere and the gradient at the large values becomes almost zero. This is known as the vanishing gradient problem. To address this problem, restricting the rectified linear units (ReLU) was introduced [28]. Glorot et al. [29] showed that the use of the ReLU activation function in the hidden layers improved the learning speed of various deep neural networks. As the ReLU activation function does not have a vanishing gradient problem and does not involve considerable computational cost, it was used as an optimizer in this study (Equation (9)).

(8) $$MinMaxScaler=\frac{x_{i}-min(x)}{\max\left(x\right)-min(x)}$$

(9) $$h(x)=\left\{\begin{array}{c} x(x>0)\\ 0(x\leq 0) \end{array}\right.$$

where *x_i_* is the *i*th data, min(*x*) is the data minimum, and max(*x*) is the data maximum; *h(x)* is the function result, and *x* is the data value.

One of the important factors in deep learning is the number of times of learning (i.e., epochs). If the number of epochs is small, learning may be insufficient; if the number of epochs is large, overfitting may occur. Therefore, an appropriate number of epochs is required. In this study, analysis was conducted 100 to 20,000 times in each environment to prevent overfitting, and a change in the loss value according to the number of epochs was confirmed. Overfitting occurred between 1000 and 1500 times for the loss value, and the learning was terminated (if it did not improve at 1000 times or more) by using the early stopping function that can end learning when the loss value does not improve.

### 2.4. RNN-LSTM Model Input Factor for Prediction of Soil Moisture

It has been previously reported that the accuracy of SM prediction can be improved by properly selecting the input variables of the model [30,31]. Selecting input variables to predict SM in soybean cultivation is an important factor that can change the performance of the model. Environmental factors for crop growth, such as T, RH, P, ST, and current soil moisture (C-SM), were used as input variables because they affect changes in future soil moisture (F-SM). T affects soil moisture reduction, P influences soil moisture increase, and RH and present SM affect both soil moisture loss and increase. Therefore, the T, RH, P (acquired by the Agricultural Meteorological Agency), ST, and SM (acquired by the soil sensor) were used as input variables to find the optimal factors for predicting future soil moisture (F-SM).

Table 2 shows a combination of several factors, such as T, RH, P, and ST; current soil moisture is the basic factor of an analysis method for finding optimal factors and combinations using soil depth (10 cm, 20 cm, and 30 cm).

### 2.5. Development of RNN-LSTM Model for Prediction of Soil Moisture and Verification of Performance

For the analysis and prediction, 56% of the entire data (13,602 values) (from 0:00 on 5 July 2020 to 10:50 on 26 August 2020) were used as learning data and 44% as verification data (6000 values). To verify the performance of the developed SM prediction LSTM model, the verification set was divided into two cases. The first set (SET I) consisted of 6000 data points from 19:30 on 26 August 2020 to 11:10 on 7 October 2020. The training data were taken between 19:30 on 26 August 2020 and 14:00 on 1 October 2020. Consequently, the data beyond the scope of the training data were taken between 14:00 on 1 October 2020 and 11:10 on 7 October 2020. The second data set (SET II) consisted of 5152 data points from 19:30 on 26 August 2020 to 14:00 on 1 October 2020. In SET II, the time series pattern of the SM in the test dataset was included in the time series patterns of SM in the training dataset. The verification data set was separated at 14:00 on 1 October because it was the reference point at which the SM began to be lower than the lowest SM among the learning data before 19:30 on 26 August.

To evaluate the SM prediction performance of the developed model, the mean square error (MSE) and coefficient of determination (R^2^) between the measured and predicted values of the model were calculated (Equations (9) and (10)).

(10) $$\mathrm{MSE}=\frac{1}{n}\sum_{i=1}^{n}{\left(y_{i}-{\ddot{y}}_{i}\right)}^{2}$$

(11) $$R^{2}=1-\frac{\sum_{i=1}^{n}{\left(y_{i}-{\ddot{y}}_{i}\right)}^{2}}{\sum_{i=1}^{n}{\left(y_{i}-\overset{-}{y}\right)}^{2}}$$

where *y_i_* is the *i*th actual value, *i* ${\ddot{y}}_{i}$ is the *i*th predicted value, $\overset{-}{y}$ is the average value of *y*, and *n* is the number of data points.

The MSE measures the accuracy of the prediction by calculating the loss function of the algorithm, and a value is averaged by taking the squared difference between the measured value of soil moisture and the predicted value of the deep learning model at a future point in time. The R^2^ evaluates the performance of the algorithm, indicating how much the actual value is calculated in regression analysis; the closer it is to 1, the better the performance will be.

## 3. Results and Discussion

### 3.1. Weather and Environmental Data

**Figure 3:**
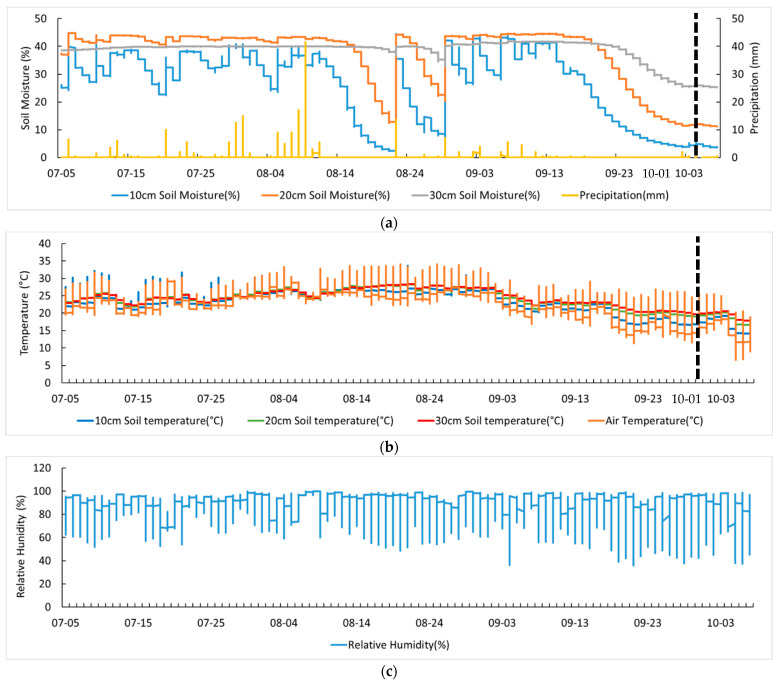
Weather and environmental time series data for soybean cultivation. (**a**) Soil moisture content time series and precipitation data by depth, (**b**) air temperature and soil temperature time series by depth, and (**c**) relative humidity time series data.

Figure 3 shows the results of measuring weather and environmental data for soybean cultivation. From the 100 days of cultivation, it rained for 47 days, was cloudy for 16 days, and was sunny for 37 days; the daily P was in the range from 0 to 101.5 mm. The T and RH at the soybean cultivation field were 23.72 °C and 82.55%, respectively. The moisture content of the soil was greatly affected by P, but the reaction rate was different depending on the depth of the soil.

### 3.2. Development of RNN-LSTM Model for Prediction of Soil Moisture Content

Using weather data (T, P, and RH) and environmental data (SM, ST), we developed a model to predict SM and selected the optimal factor. Therefore, we refined the RNN-LSTM model for SM prediction by adjusting the input variables to 2, 3, 4, and 5 for three different soil depths (10, 20, and 30 cm) at the soybean cultivation field.

#### 3.2.1. Two-Input-Factors Model: Development of RNN-LSTM Model for Prediction of Soil Moisture

In the two-input-factors model, an RNN-LSTM model that predicts SM after 10 min in soybean cultivation was developed using the SM and environmental factors as input variables, i.e., two input variables at soil depths of 10, 20, and 30 cm (Table 3). When the SM (at 10, 20, and 30 cm depths) and P were used as input variables and when the SM and RH were used as input variables, the training models were verified with SETI and showed the lowest validation loss value. As a result of developing a SM prediction model for 10 cm soil depth, the accuracy of the training model was an R^2^ of 0.999 and a loss of 0.039 when the SM and P were used as input variables. As a result of verifying with SETI, the validation loss obtained the best result, i.e., equal to 0.123. The R^2^, loss, and validation loss (SET I) were equal to 0.999, 0.016, and 0.098 at a depth of 20 cm, respectively, and 0.922, 0.01, and 7.975 at a depth of 30 cm, respectively. The accuracy of the SM was the highest at a depth of 20 cm, and the predicted accuracy tended to be lower at a depth of 30 cm. As in later models, when predicting the SM at a depth of 30 cm, it shows that SM containing learning data is predicted. However, when the SM fell below that level, the prediction performance decreased. In the process of learning the model, there were no cases where the SM did not decrease below 32.7%. Thus, it is judged that it is difficult to keep up with the trend in the case of predictions that are lower than this value. The results verified with SET II were similar to those of SET I; the validation loss was 0.1344 when the SM and P were used as an input variable at a depth of 10 cm; the validation loss was 0.1147 when the SM at a depth of 20 cm and 1.2867 when the SM at a depth of 30 cm, and P were used as input variables.

**Figure 4:**
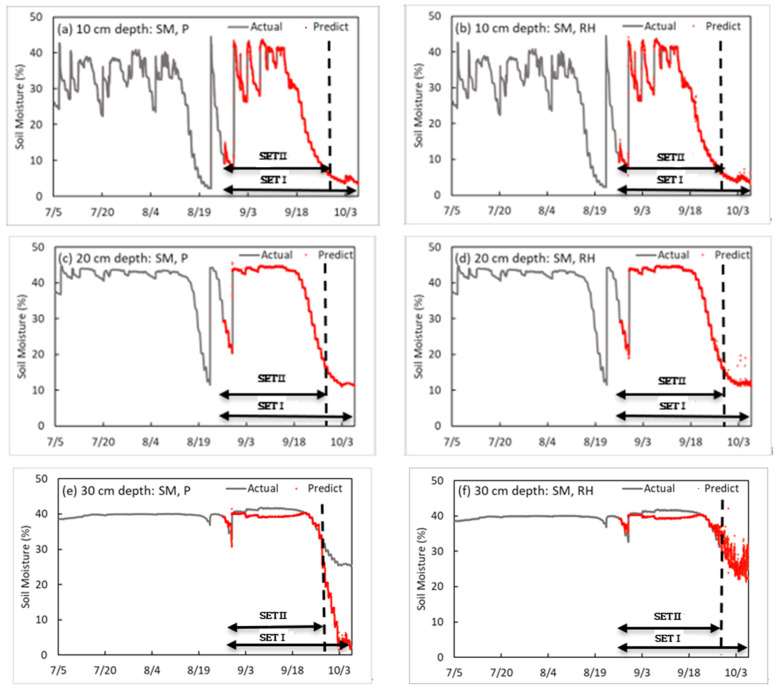
Results of the soil moisture prediction model by depth in two input factors.

**Figure 5:**
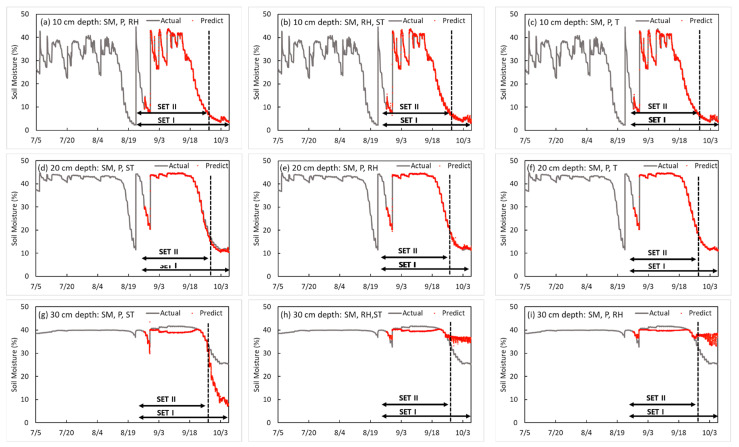
Results of the soil moisture prediction model by depth in three input factors.

**Figure 6:**
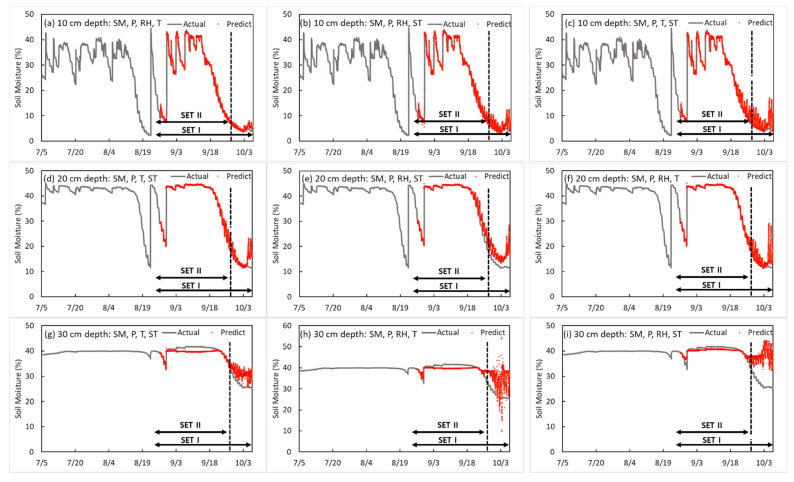
Results of the soil moisture content prediction model by depth in the four-input-factor model.

**Figure 7:**
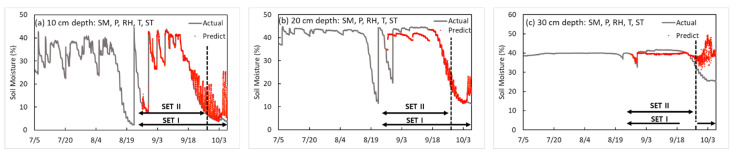
Results of the soil moisture prediction model by depth the five-input-factor model.

As a result of comparing each set, when predicting SM using two factors, the SM and P were selected as the optimal factors at each depth (10, 20, and 30 cm), which seemed to be more affected by P than RH because P is directly related to soil moisture. Next, RH was found to be affected. The result of the training model that includes RH and SM as factors at 10 cm depth were 0.998 for R^2^, 0.031 for loss, 0.238 for validation loss of SET I, and 0.275 for validation loss of SET II. At depths of 20 cm and 30 cm, the R^2^, loss, validation of loss SET I, and validation loss of SET II of the training model were 0.999, 0.06, 0.170, and 0.124 and 0.904, 0.009, 3.895, and 3.502, respectively. In the case of the verification at 20 cm and 30 cm depths, the model with the precipitation factor performed better than the model with the relative humidity factor. After RH, the accuracy increased high in the order of ST and T. Therefore, for predicting SM using two factors, the SM and P were selected as the optimal factors. Next, the SM and RH were selected. Figure 4 shows the prediction model with the best performance for each depth in two input factors. Figure 4, Figure 5, Figure 6 and Figure 7 are graphs showing 13,604 time series data measured from 12 p.m. on 5 July 2020 to 11:10 a.m. on 7 October 2020 and 6000 time series results predicted from 7:30 p.m. on 26 August 2020 to 11:10 a.m. on 7 October 2020. In the case of the prediction model using both SM and P, as shown in Figure 4a, the reason for the soil moisture being maintained at about 15% at a depth of 20 cm and less than 32% at a depth of 30 cm is that the training model does not include data showing time series characteristics; thus, the prediction error is large.

#### 3.2.2. Three-Input-Factors Model: Development of RNN-LSTM Model for Prediction of Soil Moisture

In the three-input-factor model, the RNN-LSTM model for predicting SM in soybean cultivation was developed by soil depth (10 cm, 20 cm, and 30 cm) using two optimal factors in the SET I period selected by two input factors and one of the remaining environmental factors as input variables.

Table 4 and Table 5 show the results of developing an SM prediction model by soil depth. The accuracy of the training model was determined by an R^2^ of 0.999, a loss of 0.022, a validation loss SET I of 0.105, and a validation SET II of 0.106 at a depth of 10 cm, and P and RH were used as input variables 10 min before. At depths of 20 cm and 30 cm, when the SM, P, and ST were input variables, the lowest validation loss was obtained. At a depth of 20 cm, the R^2^, loss, and validation loss SET I and SET II were 0.999, 0.067, 0.062 and 0.063, respectively; at a depth of 30 cm, they were 0.952, 0.013, 50.439, and 7.765, respectively. Similar to the two-input-factor model, in the three-input-factor model, the SM accuracy was the highest at a depth of 20 cm, and the predicted accuracy tended to decrease at a depth of 30 cm. The model obtained using the factors of SM, RH, and T at a depth of 10 cm in the three-input-factor model was the third best, and the model using T as a factor had better accuracy than using ST. Therefore, when making a model with three factors, SM, P, and RH were selected as the optimal factors, and it is concluded that the soil at a depth of 10 cm is more sensitive to external environmental factors than other depths. When predicting at depths of 20 cm and 30 cm with three factors, the use of SM, P, and ST yielded good results in both SET I and SET II verification processes. Thus, they were selected as optimal factors. The model using ST had better accuracy than using T. Therefore, unlike for a depth of 10 cm, it is inferred that the larger the depth, the more affected the inside of the soil will be. In addition, P and RH are closely related at a depth of 10 cm. For depths of 20 cm and 30 cm, SM, P, and ST were selected as optimal factors.

#### 3.2.3. Four-Input-Factor Model: Development of RNN-LSTM Model for Prediction of Soil Moisture

The four-input-factor model is an RNN-LSTM model that predicts SM by soil depth in soybean cultivation using four input variables (i.e., three optimal factors selected by three input factors and one of the remaining environmental factors). The optimal factor combination was developed in type 1 (SM, P, RH, and T), type 2 (SM, P, RH, and ST), and type 3 (SM, P, T, and ST) (shown in Table 6). As a result of developing a SM prediction model for each soil depth, SM, P, RH, and T were used as input variables at 10 cm depth; the accuracy of the training model was determined by R^2^ = 0.997, loss = 0.034, validation loss SET I = 0.678, and validation loss SET II = 0.542. At a depth of 20 cm, when SM, P, ST, and T were input variables 10 mins before, the best validation loss was obtained. At a depth of 20 cm, the R^2^, loss, validation loss of SET I, and validation loss of SET II were 0.987, 0.052, 3.620, and 1.318, respectively; at a depth of 30 cm, they were 0.956, 0.057, 5.837, and 2.883, respectively. This shows that at a depth of 10 cm, external factors, such as T and RH, were more influential than soil internal factors. In contrast, at 20 cm and 30 cm, soil internal factors, and T capable of transferring energy into the soil, were more influential than RH. Figure 6 indicates the prediction model that showed the best performance for each depth in the four-input-factor model. As shown in Figure 6, in the case of the SM + P + RH + ST prediction model, the prediction error increased after 18 September at a soil depth of 10 cm, and in the case of 4–5 October, the prediction error dramatically increased. In the case of the SM + P + RH + T prediction model, the prediction error increased when it was maintained at 15% or less at a soil depth of 20 cm.

#### 3.2.4. Five-Input-Factors Model: Development of RNN-LSTM Model for Prediction of Soil Moisture

The five-input-factor model is an RNN-LSTM model that predicts SM in the soybean cultivation field at 10 cm, 20 cm, and 30 cm soil depths using five environmental factors (SM, P, RH, T, and ST) as input variables (shown in Table 7).

According to the development of a SM prediction model by soil depth, the SM, P, RH, and ST were used as input variables for a depth of 10 cm, the accuracy of the training model was R^2^ = 0.928 and loss = 0.017. The validation loss was 20.225 for SET I, and 10.544 for SET II. At a depth of 20 cm, the R^2^, loss, and validation losses for SET I and SET II were 0.866, 0.01, 24.7641, and 26.3508, respectively. At a depth of 30 cm, the R^2^, loss, and validation losses for SET I and SET II were 0.034, 0.008, 45.1097, and 9.3472, respectively (shown in Figure 7).

### 3.3. Study of Development Results of RNN-LSTM Model for Prediction of SM and Comparison of Models

As a result of the verification of SET I and SET II, it was confirmed that the verification performance of SET II was superior to that of SET I. SET II is the result of verifying data from 19:30 on 26 August 2020 to 0:00 on 1 October 2020, and SET I is the result of verifying data from 19:30 on 26 August 2020 to 11:10 on 7 October 2020. The difference in time series data between SET I and SET II is from 1 on 1 October 2020 to 11:10 on 7 October 2020, and the ST, T, and P data for this period were confirmed. Time series data of T and ST from 1 October 2020 to 7 October 2020 showed an average decrease of 6 °C (Figure 3b). In addition, the average daily P from 4 July 2020 to 1 October 2020 and from 1 October 2020 to 7 October 2020 was 15.52 mm and 0.25 mm, respectively, thus showing a difference of 15.27 mm (Figure 3a). The weather time series patterns such as precipitation, air temperature, and soil temperature before and after 1 October were different depending on the seasonal characteristics.

When using SET I as the verification data set, the best selection (Table 8) was the SM, P, and RH factors (R^2^ = 0.999, loss = 0.022, validation loss SET I = 0.1049, and validation loss SET II = 0.1062) at 10 cm depth; SM, P, and ST factors (R^2^ = 0.999, loss = 0.067, validation loss SET I = 0.0618, and validation loss SET II = 0.0627) at 20 cm depth; and SM, P, T, and ST factors (R^2^ = 0.956, loss = 0.057, validation loss SET I = 5.8374, and validation loss SET II = 2.8828) at 30 cm depth. However, when SET II was used for validation, at 30 cm depth, the SM and P factors (R^2^ = 0.922, loss = 0.01, validation loss SET I = 7.9746, and validation loss SET II = 1.2867) were the best choices. As a result of this study, an R^2^ of 0.999 for predicting soil moisture at a depth of 10 cm was better than an R^2^ of 0.947 reported by Hong who used temperature, humidity, wind speed, insolation, precipitation, and soil temperature as input variables [32]. The prediction accuracy of R^2^ of 0.999 for soil moisture at a depth of 20 cm in this study was superior to that of an R^2^ of 0.818 reported by Yu who used temperature, sunshine hours, precipitation, wind speed, and relative humidity of the corn field as input variables [33]. The prediction accuracy of R^2^ of 0.999 for soil moisture at a depth of 30 cm in this study was better than that of an R^2^ of 0.645 reported by Qing who used soil moisture change, precipitation, and soil moisture dynamics [34]. The 10 cm soil moisture prediction using temperature, precipitation, and three depths of soil in previous studies resulted in a model accuracy of 74.64% [35]. In this study, soil moisture at 10, 20, and 30 cm depths was predicted using public weather data and soil moisture and temperature data. It was confirmed that the performance was superior compared to that in other studies.

As an optimal factor combination exists for each depth and all factors used in the experiment are included in the optimal factor combination, all factors are considered to be meaningful in the experiment. At a depth of 10 cm, because of proximity to the surface, the SM model contains P, RH, T, and ST, and external factors in the order of influence. In addition, when the ST was added to the model, the accuracy is lower than when other factors are added. In the case of 20 cm and 30 cm depths, the model using the ST performs better than when more factors are added. Therefore, it is concluded that it is affected by the internal part of the soil rather than external weather factors, except for T. As a result of the SM verification at a depth of 30 cm, the prediction accuracy was high when the pattern of the verification time series data was included in the learning data, such as SET II, but otherwise, the prediction accuracy was reduced. As a result of SM verification at a depth of 30 cm, high prediction accuracy was shown when the pattern of verification time series data was included in the learning data, such as SET II, but the prediction accuracy was lowered otherwise. In other words, in the deep learning process of the model, there was no case where the SM was not lower than 32.7%, and the prediction error increased when it was lowered below that level. Similarly, the predictive performance deteriorated when the SM fell below 11.5% at a depth of 20 cm. As this study performed the analysis using only data from 2020, it seems that the seasonal effect that changes in the second half of the data could not be kept up to date. Therefore, SET I is evaluated to be appropriate for soil prediction in areas when time series patterns are unknown, and SET II for soil prediction when time series patterns are repeated. In the future, the accuracy of the SM prediction can be improved by using various time series data patterns and time series data of a longer period [36]. In addition, the developed model is expected to be applicable to crops sensitive to moisture stress, such as red beans and corn [37].

## 4. Conclusions

In this study, the RNN-LSTM models to predict the future SM of a soybean cultivation field were developed using time series patterns of SM and weather data up to the forecasting point. First, we selected the optimal cultivation environment variable for predicting SM when cultivating soybeans and developed an RNN-LSTM model for predicting SM by soil depth. To improve the SM prediction performance, a prediction model for each depth combination was developed and verified with SET I including data outside the learning range and SET II without data. The SET I verification showed that the SM, P, and RH (R^2^ of 0.999, loss of 0.022, and validation loss of 0.105) were selected at a depth of 10 cm; the SM, P, and ST (R^2^ of 0.999, loss of 0.067, and validation loss of 0.062) were selected at a depth of 20 cm; and the SM, P, T, and ST (R^2^ of 0.956, loss of 0.057, and validation loss of 5.837) were selected at a depth of 30 cm. The SET II validation showed that SM and P (R^2^ of 0.922, loss of 0.01, and validation loss of 1.287) were the best selection at a depth of 30 cm. The characteristics of the most optimal factors according to the conditions of the learning data were investigated for the RNN-LSTM based on time series SM data. Through this study, the RNN-LSTM model was used to confirm the predictability of SM in soybean arable land, and it can be applied to predict the necessary moisture supply for crop growth. By predicting the future soil moisture, it can help in decision-making necessary for crop cultivation, such as whether to irrigate or not. Additional research will be conducted to improve the prediction performance by increasing the data acquisition period and size in the future.

## Figures and Tables

**Table 1:** Description of soil sensor SDI-12, Sentek Drill & Drop Probes, Australia.

Type	Numerical Value
Outer Probe Diameter (Top/Bottom)	30 mm/28.75 mm
Resolution	Moisture = 1:10,000/Salinity = 1:3000/Temperature= 0.3 °C
Moisture Precision	±0.03% vol
Temperature Accuracy	±2 °C @ 25 °C
Operating Temperature	−20 °C to +60 °C

**Table 2:** Details of input variables.

Soil Depth	Input Variables			
10 cm, 20 cm, 30 cm	Two Input Factors SM ^(a)^ + One factor	Three Input Factors SM + Two factors	Four Input Factors SM + Three factors	Five Input Factors SM + Four factors
	(P ^(b)^, RH ^(c)^, T ^(d)^, ST ^(e)^)	(H + P, H + ST, ST + P, H + T, T + P, ST + T)	(ST + H + P, T + H + P, T + ST + H, T + ST + P)	(P + H + T + ST)

Note: ^(a)^ SM: Soil moisture, ^(b)^ P: Precipitation, ^(c)^ RH: Relative humidity, ^(d)^ T: Air temperature, ^(e)^ ST: Soil temperature.

**Table 3:** Performance comparison of prediction model of soil moisture in the two-input-factors.

Soil Depth	Performance		Two Input Factors			
			SM ^(a)^ + P ^(b)^	SM ^(a)^ + RH ^(c)^	SM ^(a)^ + T ^(d)^	SM ^(a)^ + ST ^(e)^
10 cm	R^2^		0.999	0.998	0.378	0.996
	Loss (MSE)		0.039	0.031	0.022	0.001
	Val_loss (MSE)	SET I	0.123	0.238	47.133	1.347
		SET II	0.134	0.275	0.370	0.418
20 cm	R^2^		0.999	0.999	0.957	0.816
	Loss (MSE)		0.016	0.06	0.012	0.001
	Val_loss (MSE)	SET I	0.098	0.170	11.903	46.323
		SET II	0.115	0.124	4.425	13.555
30 cm	R^2^		0.922	0.904	0.269	0.789
	Loss (MSE)		0.01	0.009	0.008	0.025
	Val_loss (MSE)	SET I	7.975	3.895	463.175	26.258
		SET II	1.287	3.502	17.143	7.965

Note: ^(a)^ SM: Soil moisture, ^(b)^ P: Precipitation, ^(c)^ RH: Relative humidity, ^(d)^ T: Air temperature, ^(e)^ ST: Soil temperature.

**Table 4:** Performance comparison of the prediction model of soil moisture in the three-input-factors I.

Soil Depth	Performance		Input Factor		
			SM ^(a)^ + P ^(b)^		
			RH ^(c)^	T ^(d)^	ST ^(e)^
10 cm	R^2^		0.999	0.999	0.981
	Loss (MSE)		0.022	0.035	0.032
	Val_loss (MSE)	SET I	0.105	0.247	5.569
		SET II	0.106	0.195	2.704
20 cm	R^2^		0.999	0.998	0.999
	Loss (MSE)		0.045	0.098	0.067
	Val_loss (MSE)	SET I	0.122	0.476	0.062
		SET II	0.078	0.436	0.063
30 cm	R^2^		0.664	0.419	0.952
	Loss (MSE)		0.023	0.035	0.013
	Val_loss (MSE)	SET I	25.893	30.989	50.439
		SET II	9.110	9.185	7.765

Note: ^(a)^ SM: Soil moisture, ^(b)^ P: Precipitation, ^(c)^ RH: Relative humidity, ^(d)^ T: Air temperature, ^(e)^ ST: Soil temperature.

**Table 5:** Performance comparison of the prediction model of soil moisture in the three-input-factors II.

Soil Depth	Performance		Input Factor		
			SM ^(a)^ + RH ^(c)^		
			P ^(b)^	T ^(d)^	ST ^(e)^
10 cm	R^2^		0.999	0.984	0.999
	Loss (MSE)		0.064	0.032	0.031
	Val_loss (MSE)	SET I	0.105	4.728	0.224
		SET II	0.106	2.668	0.205
20 cm	R^2^		0.999	0.995	0.996
	Loss (MSE)		0.037	0.024	0.281
	Val_loss (MSE)	SET I	0.122	1.191	1.834
		SET II	0.078	0.535	0.528
30 cm	R^2^		0.664	0.550	0.850
	Loss (MSE)		0.051	0.023	0.011
	Val_loss (MSE)	SET I	25.893	25.428	22.297
		SET II	9.110	8.166	7.338

Note: ^(a)^ SM: Soil moisture, ^(b)^ P: Precipitation, ^(c)^ RH: Relative humidity, ^(d)^ T: Air temperature, ^(e)^ ST: Soil temperature.

**Table 6:** Performance comparison of the prediction model of soil moisture in the four-input-factor-model.

Soil Depth	Performance		Input Factor		
			SM ^(a)^ + P ^(b)^ + RH ^(c)^ + T ^(d)^	SM ^(a)^ + P ^(b)^ + RH ^(c)^ + ST ^(e)^	SM ^(a)^ + P ^(b)^ + T ^(d)^ + ST ^(e)^
10 cm	R^2^		0.997	0.990	0.981
	Loss (MSE)		0.034	0.024	0.077
	Val_loss (MSE)	SET I	0.678	2.770	5.924
		SET II	0.542	1.647	3.141
20 cm	R^2^		0.974	0.982	0.987
	Loss (MSE)		0.031	0.020	0.052
	Val_loss (MSE)	SET I	5.669	8.768	3.620
		SET II	1.162	4.573	1.318
30 cm	R^2^		0.481	0.237	0.956
	Loss (MSE)		0.01	0.09	0.057
	Val_loss (MSE)	SET I	21.387	33.792	5.837
		SET II	8.308	6.960	2.883

Note: ^(a)^ SM: Soil moisture, ^(b)^ P: Precipitation, ^(c)^ RH: Relative humidity, ^(d)^ T: Air temperature, ^(e)^ ST: Soil temperature.

**Table 7:** Performance comparison of the prediction model of soil moisture in the five-input-factor-model.

Soil Depth	Performance		Input Factor
			SM ^(a)^ + P ^(b)^ + RH ^(c)^ + T ^(d)^ + ST ^(e)^
10 cm	R^2^		0.928
	Loss (MSE)		0.017
	Val_loss (MSE)	SET I	20.225
		SET II	10.544
20 cm	R^2^		0.866
	Loss (MSE)		0.01
	Val_loss (MSE)	SET I	24.764
		SET II	26.351
30 cm	R^2^		0.034
	Loss (MSE)		0.008
	Val_loss (MSE)	SET I	45.110
		SET II	9.347

Note: ^(a)^ SM: Soil moisture, ^(b)^ P: Precipitation, ^(c)^ RH: Relative humidity, ^(d)^ T: Air temperature, ^(e)^ ST: Soil temperature.

**Table 8:** Results of soil moisture best prediction model by each CASE.

Factors		R^2^	Loss (MSE)	Val_Loss (MSE)	
				SET I	SET II
10 cm	SM,P	0.999	0.039	0.123	0.134
	SM, P, RH	0.999	0.022	0.105	0.106
	SM, P, RH, T	0.997	0.034	0.678	0.542
	SM, P, RH, T, ST	0.928	0.017	20.225	10.544
20 cm	^(a)^ SM, ^(b)^ P	0.999	0.016	0.098	0.115
	SM, P, ^(e)^ ST	0.999	0.067	0.062	0.063
	SM, P, ^(d)^ T, ST	0.987	0.052	3.620	1.318
	SM, P, ^(c)^ RH, T, ST	0.866	0.01	24.764	26.351
30 cm	SM, P	0.922	0.01	7.975	1.287
	SM, P, ST	0.952	0.013	50.439	7.765
	SM, P, T, ST	0.956	0.057	5.837	2.883
	SM, P, RH, T, ST	0.034	0.008	45.110	9.347

Note: ^(a)^ SM: Soil moisture, ^(b)^ P: Precipitation, ^(c)^ RH: Relative humidity, ^(d)^ T: Air temperature, ^(e)^ ST: Soil temperature.

## Data Availability

Not applicable.
